# Three Years of African Swine Fever in South Korea (2019–2021): A Scoping Review of Epidemiological Understanding

**DOI:** 10.1155/2023/4686980

**Published:** 2023-02-23

**Authors:** Jun-Sik Lim, Mathieu Andraud, Eutteum Kim, Timothée Vergne

**Affiliations:** ^1^IHAP, Université de Toulouse, INRAE, ENVT, Toulouse 31300, France; ^2^Anses, Ploufragan-Plouzané-Niort Laboratory, Epidemiology, Health and Welfare Research Unit, Ploufragan 22440, France; ^3^College of Veterinary Medicine and Institute of Veterinary Science, Kangwon National University, Chuncheon 24341, Republic of Korea

## Abstract

African swine fever (ASF) is a highly contagious viral disease in domestic pigs and wild boar that causes tremendous socioeconomic damage in related industries. In 2019, the virus emerged in South Korea, which has since reported 21 outbreaks in domestic pig farms and over 2,600 cases in wild boar. In this review, we synthesize the epidemiological knowledge generated on ASF in South Korea during the first three years of the epidemic (2019–2021). We searched four international and one domestic Korean database to identify scientific articles published since 2019 and describing ASF epidemiology in South Korea. Fourteen articles met our selection criteria and were used to synthesize the origin of ASF in South Korea, the risk factors of disease occurrence, the effectiveness of the surveillance and intervention measures that were implemented, and the viral transmission dynamics. We found that timely intensive surveillance and interventions on domestic pig farms successfully blocked between-farm transmission. However, in wild boar, the ASF virus has spread massively towards the south primarily along the mountain ranges despite ongoing fence erection and intensive depopulation efforts, endangering domestic pig farms across the country. The current devastating epidemic is suspected to be the consequence of an ASF control strategy unaligned to the epidemiological context, the challenging implementation of control measures hindered by topological complexities, and inappropriate biosecurity by field workers. To improve our understanding of ASF epidemiology in South Korea and enhance disease management, future research studies should specify the ecological drivers of disease distribution and spread and devise effective control strategies, particularly in relation to Korean topography, and the latent spread of the virus in wild boar populations. Additionally, research studies should explore the psychosocial factors for ASF management, and develop tools to support evidence-based decision-making for managing ASFV in wild boar.

## 1. Introduction

African swine fever (ASF), caused by the African swine fever virus (ASFV) and listed as a notifiable disease by the World Organisation for Animal Health (WOAH), is a highly contagious and devastating disease in domestic pigs (*Sus scrofa domesticus*) and wild boar (*Sus scrofa*) [1]. ASFV is a large, enveloped, double-stranded DNA virus in the *Asfarviridae* family. A total of 24 genotypes have been identified to date, principally in Africa, among which genotype II is responsible for the current ASF epidemics in Europe and Asia [2]. Infected hosts present clinical expressions, including generalized haemorrhagic fever, diarrhoea, and vomiting, with a case fatality rate close to 100% in both pigs and wild boar. ASF management is highly challenging due to its non-specific symptoms delaying the detection in the early stage of an epidemic [1], the co-circulation of the virus in domestic and wild compartments, spillover between two host species, interconnected environmental drivers for ASF epidemiology and wild boar ecology [3], and psychosocial factors for ASF management [4]. There is no effective vaccine or treatment.

In Asia, following the emergence of the virus in China in 2018, several Asian countries including Vietnam, Cambodia, Laos, Myanmar, Indonesia, India, North Korea, and South Korea have each, in turn, reported ASF [2, 5]. With the exception of South Korea, the countries have reported ASF cases primarily in domestic pig farms. However, the disease burden in wild boar populations is likely underestimated due to insufficient surveillance [6].

In South Korea, concerns rose over the risk of ASF introduction from neighbouring affected countries in 2018 following the detection of ASFV in pig products confiscated from travellers from Shenyang, China, where the first ASF case in domestic pigs in China was reported [7]. To prevent ASFV introduction, the Korean authorities implemented measures such as restrictions on travellers from not taking pig products from affected zones. Despite these efforts, the first domestic case was reported in a pig farm located in the northwest region near the border with North Korea in September 2019 [8–10]. In domestic pig farms, 14 cases were reported within 23 days, followed by 2 cases, and 5 cases in domestic pig farms in each of the years 2019, 2020, and 2021, respectively [11]. In wild boar, since the first report of an ASF-positive carcass in October 2019 in the northwest region near the border, more than 2,600 cases have been reported, with the disease spreading to the south and east [12].

The control measures implemented in the domestic compartment included the slaughtering of pigs on ASFV-positive farms and the preventive culling of herds within a 500 m radius. In addition, a regional and national level ban on the circulation of livestock vehicles was implemented immediately after the ASF report from domestic pig farms, as well as active surveillance for epidemiologically linked farms [9, 10, 13, 14]. In particular, due to the risk of ASF outbreaks, the pigs on around 250 domestic pig farms in ASF-affected counties and neighbouring counties have been pre-emptively slaughtered [15]. Since October 2020, the South Korean government designated ASF-affected counties and their neighbouring counties as “Districts Subject to Intensive Disease Control for African Swine Fever” and mandated specified biosecurity facilities on the farms in the districts [16]. Moreover, since 2021, the government divided the entire country into 16 subregions based on the livestock vehicle network, and restricted the movement of live pigs and manure within each subregion [17].

Specific surveillance and control policies also were conducted for ASF in wild boar. Active carcass search and removal, hunting, and trapping were carried out by government-employed people who sampled animals to be tested for ASFV. Three types of fences were built considering the wild boar movement: the first and second fences enclose the areas where one or more ASF-positive wild boar or carcasses were reported, covering a radius of 1–2 km and 5–10 km, respectively. The third was erected 20–30 km from the second, traversing the Korean peninsula, to prevent wild boar from migrating south [9]. Whenever an ASF case was reported beyond the third fence, the authorities built fences further south to enclose the newly infected regions [18]. South Korea is considered to have implemented control policies with a relatively high level of performance among Asian countries due to successful control of ASF in domestic pig farms and intensive measures in wild boar [6, 9, 10].

Many research studies have contributed to a better understanding of the epidemiology of ASF. However, the majority of them have focused on the European context [19–22]. Given the diversity of ASF epidemiological patterns [23], the epidemiological knowledge derived from the unprecedented ASF situation in South Korea needs to be synthesized. This could be useful to design surveillance and control measures for South Korea and other affected countries. This scoping review, which covers primary research articles focused on ASF epidemiology in South Korea, synthesizes current knowledge and highlights future research directions.

## 2. Methods

### 2.1. Literature Search

In this study, we followed the guidelines of the PRISMA-ScR (Preferred Reporting Items for Systematic Reviews and Meta-Analyses Extension for Scoping Reviews) statement for scoping reviews [24]. PubMed, Scopus, Embase, and Web of Science were screened to find all relevant epidemiological studies for ASF in South Korea published in peer-reviewed journals. The Boolean query that was used to screen the titles and abstracts of the articles listed in the four databases was defined using the following terms (“Korea”(tiab) AND “African swine fever”(tiab)). We restricted the publication year to studies published since 2019, which corresponds to the ASF epidemic period in South Korea. No restriction was imposed on language. The search was conducted once in January 2022 and again in August 2022. Moreover, to identify the research studies published in Korean domestic peer-reviewed journals, we screened the “Korea Citation Index” (KCI) (https://www.kci.go.kr/kciportal/main.kci?locale=en), which is a database of information from journals operated by Korean academic societies, papers, and references. The terms “African swine fever” and “아프리카 돼지열병” in the abstract and title were used to screen KCI. The citation manager program Endnote (https://www.endnote.com/, accessed January 2022) was used to import and manage the records.

### 2.2. Literature Selection

A publication was considered eligible for inclusion if it reported primary research on ASF epidemiology in South Korea, meaning a study where the author(s) collected and/or analysed data to conduct a risk analysis of ASFV introduction into South Korea, identify risk factors for disease occurrence and its spatiotemporal distribution, assess the surveillance and intervention measures implemented, or unravel viral transmission dynamics. We excluded articles that only focused on the description of the epidemiological situation and the surveillance and intervention measures implemented, as well as the ones that did not reflect the Korean epidemiological context. In this review, for the sake of reliability and scientific objectivity, we only included articles published in peer-reviewed journals, purposively excluding grey literature, including government reports and news articles from South Korea.

Literature selection was performed by the first author (JSL) and subsequently validated by another author (EK). If the title and abstract were not sufficiently clear to exclude the record, the reference was kept for the secondary screening. The records that passed the primary screening were subsequently assessed for eligibility by applying the same inclusion and exclusion criteria to the full text. Conflicts of opinion between the two reviewers at each screening were discussed until a consensus was reached.

### 2.3. Data Extraction

Extracted information included publication information (year, author, and title), species (wild boar, domestic pig, pig product, and arthropod), research question (ASFV introduction into South Korea, surveillance and intervention effectiveness, risk factor identification and distributions, and transmission dynamics), detailed purpose, main results, and study limitations as addressed by the authors. Data extraction was performed by the first author (JSL) and validated by another author (EK). A spreadsheet frame was developed to store the extracted information from the selected records. The number of selected records was plotted according to the year, and species. The main results that describe ASF epidemiology in South Korea were summarized and presented as a narrative structured by research questions.

## 3. Results

### 3.1. Screening and General Characteristics of Selected Records

**Figure 1:**
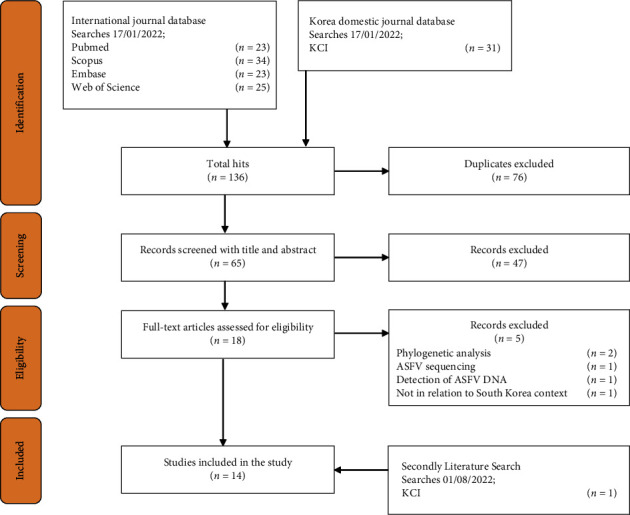
Screening and selection of articles. KCI: Korea Citation Index.

Of the 105 records extracted from PubMed, Scopus, Embase, and Web of Science and the 31 records from KCI, 76 were duplicates. Therefore, 65 records were kept for screening (Figure 1). The first screening process on the title and abstract excluded 47 records. During the eligibility assessment on the full text, five other records were excluded. These five records focused on ASF in South Korea but did not address ASF epidemiology; one record addressed ASFV transmission dynamics within a domestic pig farm using a mechanistic modelling approach where the fitted outbreak data and epidemiological parameters included were not specific to the Korean context; two records described the phylogenetic analysis of ASFV in domestic pigs and wild boar; and the remaining two records addressed ASFV sequencing and detection of ASFV DNA, respectively. In the second literature search, one additional article published in a Korean domestic journal was included [25]. A total of 14 records, with 10 coming from international databases and four from KCI, were selected as eligible for data extraction.

**Figure 2:**
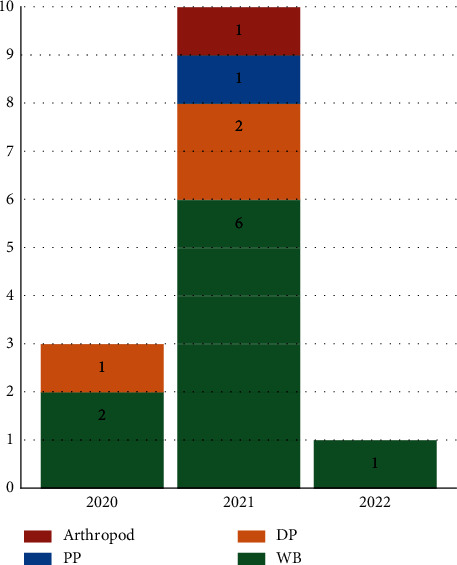
Distribution by published year(s) for each main epidemiological unit of focus in the 14 articles selected for the scoping review; PP: pig products; DP: domestic pigs; and WB: wild boar.

All 14 of the selected articles focused on ASFV genotype II reported in the period from 2019 to 2021, and were published between 2019 and 2022 (Table 1). The number of records has risen substantially over time, with four publications in 2020, eleven in 2021, and one in 2022 (at the time of the search). The host or material of primary interest most represented in the selected records was wild boar (nine records, 64.3%), followed by domestic pigs (three, 21.4%), pig products (one, 7.1%), and arthropods (one, 7.1%) (Figure 2).

### 3.2. Where did the ASF Virus Come from?

Three records focused on the introduction routes of ASFV into South Korea, investigating human-related activities and the role of wild boar [26, 28, 32]. Studies of human-related activities have focused primarily on pig products and live pig imports due to the unique characteristics of South Korea, which can be considered as an island due to the tight restrictions on limited interactions across its sole land border with North Korea. Legal imports of live pigs and pork products should be considered as a less likely route of introduction, as suggested by a probabilistic risk assessment framework that revealed that the risk of ASFV introduction through these routes can be considered negligible thanks to the policy of banning imports of pig products from ASF-affected countries [28]. The authors emphasized that their risk assessment framework was not comprehensive, preventing a formal assessment of the relative probability of introduction through different human-mediated routes, such as illegal imports.

North Korea is the only country that is geographically connected to South Korea, and there are few human-mediated interactions between the two countries. The first ASF case in North Korea was reported before the detection of ASFV emergence in South Korea, spurring an unresolved debate about the likelihood of ASFV introduction via wild boar from North Korea. This hypothesis is supported by epidemiological data since a spatial model of ASF case distribution highlighted the proximity to North Korea as a major risk factor for the occurrence of ASF in wild boar during the early phase of the epidemic (October 2019–Jan 2020) [32]. On the one hand, the likelihood of wild boar crossing the border is often considered negligible due to the presence of fences along the national boundary [26]. Therefore, the hypothesis of human-mediated introduction was stressed as being the most realistic, although exact mechanisms could not be identified [26].

### 3.3. Risk Factors and Spatiotemporal Distributions

Identifying risk factors is essential to determine favourable conditions for ASF introduction and transmission in susceptible host populations and to design risk-based surveillance strategies. While ASF risk factors have been studied in both wild boar and domestic pigs in several settings [39], in South Korea, only one selected article investigated risk factors in wild boar.

As mentioned previously, the proximity to North Korea was identified as a strong risk factor for disease occurrence in wild boar during the early phase of the epidemic (Oct 2019–Jan 2020) [32]. Following the early phase of the epidemics, during the following period (Jan 2020–Apr 2020), spatial proximity to a region reported affected during the previous period explained well the spatial distribution of ASF-positive wild boar. The authors concluded that the epidemiological situation evolved to mainly comprise within-country transmission, rather than potential reintroduction from North Korea [32]. Furthermore, it was suggested that the wild boar population had been disturbed as a result of surveillance and population control efforts [32]. Environment characteristics that might relieve symptoms caused by ASFV infection were identified as risk factors for ASF-positive carcass distribution and were suggested as surveillance and intervention targets [32].

According to the spatiotemporal clusters identified by one study [31], at least two regions (northwest and north) were possible sources of the current ASF outbreaks in wild boar in South Korea, each of which became seeds for the invasion spreading into the southwest and southeast, respectively, while another study suggested continuous risks from North Korea during the early phase of the ASF epidemic [32]. Although the expansion of ASF toward the southwest appears to be contained [31], concerns have been raised regarding a spread toward southeast regions along geographical fast-tracks. The forested Taebaek mountains, which run from north to south and are a highly favourable habitat for wild boar, are located in the eastern region of the Korean peninsula, providing a reservoir zone as a source of ASFV [25, 32, 36]. In addition, due to mountain elevations above 1,000 m on average, carcass detection and depopulation are challenging, which favours persistence in the environment. ASFV is thus highly likely to spread south more rapidly through these mountain ranges [32, 36]. The predictive study of ASF in wild boar in Gangwon province, where most of the Taebaek mountain ranges are located, showed that a high risk of ASF-positive carcasses is distributed along the mountain ranges in the western and southwestern parts of the province, whereas the risk of ASF-positive hunted wild boar is distributed in western regions of the Taebaek mountain range in the central part of the province [25].

Over the two years, ASF in wild boar has shown consistent seasonality, with peaks in the winter and spring, even though ASF has expanded to southeast regions. About 70 cases of ASF-positive carcasses have been reported on average per month, yet between January and April, this figure rises to over 90 cases per month. In particular, 185 and 168 cases were reported, respectively, in March 2020 and February 2021, although it is not clear whether this seasonality is due more to disease dynamics or different surveillance intensities [25]. In addition, spatiotemporal high-risk clusters of the ASF-positive carcass, which were considered to be less impacted by the imperfect detection sensitivity, were also primarily found in the winter and spring. Active and interactive behaviours of wild boar during the mating season (November∼January) were hypothesized to be associated with this seasonality [36].

### 3.4. Transmission Dynamics

In South Korea, there were a total of 27 ASF cases in domestic pig farms, with 14 cases occurring successively over 23 days in the early stages (September 2019–October 2019) and the remaining 13 cases occurring intermittently until October 2022. In wild boar, over 2,600 cases have been continuously detected in the country since October 2019. Nine articles investigated the transmission dynamics in domestic pigs or wild boar by either estimating transmission parameters or simulating disease dynamics.

#### 3.4.1. Domestic Pig Farms

Like other livestock infectious diseases [40, 41], the movement of vehicles contributed to the between-farm transmission of ASFV. For the 14 outbreaks occurring successively, livestock vehicles played a role in between-farm transmission; the transmission rate (*β*) through the transit of a potentially contaminating vehicle was estimated to be 53.9 × 10^−4^/visit, increasing the risk by 11.1 compared with a farm that was not visited by such a vehicle. Moreover, Yoo et al. [35] showed that vehicle movements accounted for 41.2% of ASF-positive farms. In particular, farms in the southwest regions, where vehicle networks are very dense, were found to be mainly infected through vehicle movement. Moreover, by estimating the relationship between spreading potential and vehicle visits, it was possible to formulate guidelines on contact rates between farms. To contain epidemics in domestic pig farms, the average number of vehicles visiting a farm in a day and the average number of farms visited by a vehicle in a day should be less than 1.3 [35].

Spillover from wild boar populations to domestic pig farms was identified as contributing to ASF dissemination in the country. A modelling study representing ASF dynamics in domestic pig farms with livestock vehicle networks and ASF-positive wild boar cases found that the latter accounted for 24% of ASF occurrences on farms. The contribution of wild boar was expected to be larger than estimated due to undetected ASF-positive wild boar, partially quantified as a background risk accounting for 35% of ASF-positive farms [35].

#### 3.4.2. Wild Boar

It is considered that ASFV self-sustains within the wild boar population in South Korea. Indeed, during 2019–2021, the national level time-varying reproduction number (*R*_*t*_) of ASF in wild boar, defined as the expected number of cases directly infected by one case in a population at a given time point, was estimated as ranging from 1.39 to 4.82, which indicates a self-sustaining cycle [33]. Moreover, ASF spreading potential was identified as heterogeneous and dependent on environmental features, as suggested by the research that estimated *R*_0_ in spatiotemporal clusters [36]. To lessen the biases caused by the heterogeneous intensity of surveillance, the high-risk spatiotemporal clusters were identified because surveillance had been intensified in the high-risk areas. For the 22 clusters detected, the estimated *R*_0_*s* ranged from 1.11 to 2.37, and they were positively associated with the habitat suitability index for wild boar produced by environmental factors. Moreover, the spreading potential at the cluster level has increased as the high-risk clusters of ASF cases have been detected in the Taebaek mountains over time. Given the contrary result of the national level *R*_*t*,_ which decreased over time from 2.94 to 2.00 between 2019 and 2020, [33], these findings suggested that ASFV has primarily expanded in forests and mountains where environments are favourable to wild boar [36]. A seasonal pattern also was identified: the *R*_*t*_ was 3.82 and 4.82 in the winter and summer, respectively, which is significantly higher than the *R*_*t*_*s* of 1.39 and 2.21 estimated for the spring and fall.

Although the indirect transmission by ASF-positive wild boar carcasses has been regarded as comparable to the direct transmission by ASF-positive live wild boar in various contexts [42], the contribution of indirect transmission in South Korea is yet to be quantified. A simulation study, which incorporated the ecological features of the wild boar population in South Korea, quantified the transmission rate (*β*) for direct contact in wild boar at 0.016/week, consistent with previous studies [38]. However, the estimated *β* due to indirect contacts with ASF-positive wild boar carcasses could not be differentiated from the estimated *β* due to direct contacts, which might be because the model did not incorporate the infection pressure from environments contaminated by an ASF-positive carcass or was too complex in comparison to the data [38].

While spillover of ASFV from domestic pigs to wild boar was identified as a key pathway to establish and promote ASFV circulation in wild boar populations in Asian countries [39, 43], the situation in South Korea is less clear [31, 35]. On the one hand, the likelihood of transmission from domestic pig herds to wild boar is considered negligible because ASFV was estimated to be circulating in wild boar populations before it was detected in pig farms. North Korea already reported ASF in wild boar before the emergence of ASFV in South Korea. The larger number of ASF cases in wild boar compared to pigs furthermore suggests that the virus can circulate in a sustainable manner in wild boar populations without the need for regular reintroductions from domestic pig farms [35]. Moreover, North Korea previously reported the disease when the first wild boar case was identified in the Korean Demilitarized Zone, where no farms or civilians were present. On the other hand, some authors suggested several spillover pathways to the wild boar population. Following the discovery of outbreaks in wild boar near military camps, it was reported that some geographically isolated camps had fed food waste to wild boar despite official restrictions on such practices. Inadequate culling procedures for ASF-positive pig farms also were suggested as the source of ASFV in wild boar, including burying slaughtered pigs and blood leakages from slaughtered ASF-positive pigs that were accessible to wild boar [31].

Several studies have contributed to understanding the spatial progression of ASF in wild boar in Europe [43, 44]. ASFV spatial invasion was categorized as wild boar-mediated transmission and viral translocation, primarily represented as short-distance and long-distance dispersal [43, 45]. In South Korea, where ASFV in wild boar has invaded from the north to the south, the characteristics of the invasion show some similarities with the EU context, but have different implications. The short-distance dispersal by wild boar was slow in South Korea, which is consistent with the EU context; the infection pressure on wild boar in neighbouring habitats was lowered to approximately 15%, compared to the infection pressure on individuals in the same geographical habitat, which was likely due to the fact that wild boar rarely interact with other boars in different habitats in South Korea [38]. The invasion was predicted to occur predominantly through forests and mountains, which raised the concern that ASFV would be disseminated throughout the country, 70% of which is covered by forests and mountains. In particular, the mountain ranges in the eastern regions of the Korean peninsula could be the primary spreading route to the south [37]. Human activities were considered to be factors for long-distance spread, as represented by the cases in the interconnected and military areas along the border, and an ASF case in wild boar reported over 30 kilometres away from the previous case [25, 26]. In particular, hunting activities were incriminated, as evidenced by ASFV DNA-positive samples from hunting vehicles, hunting dogs, hunting gear, soil from hunting areas, and untrained hunters who did not follow biosecurity measures [26]. Financial incentives for hunting and carcass detection encouraged field actors with poor biosecurity awareness to travel inside and outside fenced areas in order to participate in surveillance activities. Inappropriate biosecurity measures, therefore, may have contributed to the spread of ASF across the fences [31].

#### 3.4.3. The Role of Arthropods

Alongside direct and indirect transmission of ASFV between the *Suidae* family, blood-feeding arthropods can affect ASF dynamics. As in the EU context [46, 47], *Ornithodoros* spp. ticks, the main vector for ASF transmission in Africa, were not identified in South Korea [48]. The epidemiological focus in South Korea, therefore, has been on mechanical vectors, such as *Stomoxys calcitrans*, that are suspected to contribute to virus spread [49]. During the first 14 outbreaks of ASF in domestic pig farms, 28,729 arthropods were collected from the affected farms and their surroundings and tested by real-time polymerase chain reaction [34]; none of the specimens were found positive. The authors discussed that, due to immediate control measures, there was a limited possibility that arthropods were exposed to ASFV, but a vector-borne contribution to the transmission could not be ruled out [34].

### 3.5. Effectiveness of Surveillance and Intervention Strategies

#### 3.5.1. In Domestic Pigs

Among the 27 cases detected in domestic pig farms, only the management response to the first 14 outbreaks was evaluated. It was shown to have been successful in terms of breaking the transmission links. Indeed, the ASF detection and intervention measures in domestic pig farms were timely. First, among these 14 ASF-positive farms, 11 cases were reported by the farmers early in the infection cycle, when less than four pigs showed clinical signs such as pyrexia and anorexia [27]. The pigs in pigsties other than where clinically affected pigs were located also always tested negative, suggesting that the diffusion of ASFV was restricted to pigsties where ASF-positive pigs were detected for the first time on the farm [27, 30]. In addition, no animal in family owned and consigned farms was detected as positive [30]. The active surveillance system that was put in place in farms that were epidemiologically linked with outbreaks proved to be effective as it allowed the detection of the remaining three ASF-positive farms among the first 14 outbreak farms before the occurrence of any symptoms [30].

#### 3.5.2. In Wild Boar

Beyond the well-known difficulties involved in the implementation of effective strategies to control infectious diseases in wildlife [50], the context in South Korea presents unique surveillance and intervention challenges. The government has installed three types of fences, two of which cover a radius of 1–2 km and 5–10 km from where ASF-positive wild boar have been newly reported [9]. The third one (national fencing), which crosses the Korean peninsula, has been built to enclose the newly infected regions in an attempt to restrict wild boar movements and prevent the spatial dissemination of the virus (as of 11/03/2022) [9]. In addition, drastic measures have been implemented to reduce wild boar density and improve biosecurity practices in affected areas for field workers such as military and hunters. [9, 10].

Like other settings, the surveillance of wild boars in South Korea has limitations. After ASFV was first reported in a domestic pig, surveillance efforts for wild boar were strengthened starting from 22 September 2019, and began to detect ASFV in wild boars within 17 days after the index case in domestic pigs, suggesting that ASFV had been previously underreported in the country [35]. Moreover, the landscape characteristics of South Korea have posed substantial challenges to the implementation of surveillance activities in wild boar. Indeed, landscapes that are difficult to access, including mountains, forests, and areas with low human population density, make it difficult to detect carcasses. In addition, the border with North Korea, where a substantial number of ASF-positive wild boar were detected, is a land mine zone where only soldiers are permitted to search for wild boar carcasses [26, 32]. Surveillance in such environments is certainly not optimal and these areas represent an ecological reservoir for ASFV in wild boar, and potentially are a continuous source of virus diffusion [32]. Other environmental characteristics were shown to improve the detectability of ASF-positive carcasses, such as areas with high habitat suitability for wild boar, or cool and wet areas which might alleviate their symptoms [32]. Due in large part to these contexts, the detection sensitivity in wild boar was shown to be highly heterogeneous both in time and space, as shown by the research that applied a zero-inflated Poisson model [32]. The authors showed that in the early phase of the epidemic (Oct 2019–Jan 2020), only 49% of affected 25 km^2^ hexagons had at least one detected positive wild boar and that this proportion increased to 73% in the second phase (Jan 2020–Apr 2020).

Fencing is designed to create artificial landscape fragmentations to limit the geographical spread of pathogen and transmission events. Placement and timing are considered key elements of fencing [51]. However, it has been shown that these elements varied in each of the ASF-affected counties in South Korea in relation to the first and second fences. Some counties disagreed with the fencing and thus delayed their decisions [31]. In addition, cold environments made the immediate building of the first and second fences challenging. Furthermore, some fences have openings, leaving corridors to uninfected areas. Some authors stated that these enabled ASFV to cross the fence [31]. While the effects of fences on preventing the disease from spreading in wildlife are questionable [51], they were shown to slow the spread in South Korea. The third fencing (national fencing) was found to decrease the infection pressure on individuals in the neighbourhood habitat by 47% compared to the infection pressure on individuals in the same geographical habitat [38]. The authors suggest that it might be used as a temporary measure to buy time. Environmental and anthropogenic fragmentations such as rivers and highways were shown to have a higher effect, decreasing the infection pressure by 65% [38].

Host density is a key parameter in disease dynamics in wildlife [50]. Thus, ASF-affected countries, including Belgium and the Czech Republic, have culled wild boar in an effort to control ASF in the peripheral area of the buffer zone surrounding the reported area [3]. Following their strategies, the Korean government has implemented depopulation measures such as trapping and hunting since the first report of ASF in wild boar. However, some researchers have argued that hunting activities induced paradoxical consequences in wild boar ASF management in South Korea [31] which raise the risk of disease spread by inducing unanticipated behavioural changes in the wildlife population [52]. Some authors argued that Paju, one of the affected counties in South Korea, suffered from this paradoxical effect, reporting numerous cases when the depopulation measures changed from silent culling (trapping and army snipers) to non-silent culling (hunting). In contrast, Cheorwon, which maintained silent culling, did not experience paradoxical consequences [31]. Similarly, the depopulation of wild boar in the fenced area without considering the buffer area might contribute to causing animals to escape from the fenced area and thus transmit ASFV outside the fence [31].

In infectious diseases in wildlife, a population threshold value for the persistence of infectious diseases is a key concept for building control policies. The Korean government estimated the Korean wild boar population to be 300,000 and decided to reduce this by 33%, that is to kill 100,000 wild boar across the country in 2019 [29]. However, a simulation study showed that the 33% annual culling rate would not be enough to reduce the wild boar population in the long term [29]. The authors argued that a 33% annual culling rate would reduce the wild boar population for only two to three years, and then the population would grow and surpass the initial population. Long-term effect policy calls for a culling rate of 75% annually over a period of more than three years, as shown in the simulation [29]. Furthermore, less than half of the targeted number of animals were actually killed throughout the planned period for the entire country [31]. In addition, the recalculated density based on the animals actually culled in some ASF-affected regions was 10 animals per square kilometre, which is much higher than the government estimation and suggests that the wild boar population had been heavily underestimated [31].

The biosecurity measures for field workers were an area of concern. For example, biosecurity education did not extend to the military, wildlife experts, and non-official hunters, and was instead confined to government officers and official hunters. Furthermore, there have been reports that official hunters sampled detected carcasses without wearing personal protective equipment [31]. Activities of the public, such as local festivals and military activities in the affected counties, were mentioned as potential routes for spreading. In addition, each ASF-affected county had different levels of biosecurity efforts, ranging from a few procedures in affected areas to active participation [31].

## 4. Discussion

In this scoping review, we identified 14 studies that described epidemiological aspects of ASF in South Korea. They addressed ASFV introduction into the country, risk factors, spatiotemporal distribution, and disease transmission dynamics as well as surveillance and intervention strategies. These studies proposed several hypotheses concerning routes of ASFV introduction, none of which could be rejected. A first temporal cluster of 14 cases in domestic pigs likely related to the transit of contaminated vehicles and wild boar were successfully contained with timely and intensive control measures. After the first report on a domestic pig farm, the surveillance of wild boar was strengthened, resulting in the detection of a very large number of ASF-positive carcasses and live animals. However, the surveillance efficacy was highly dependent on landscape characteristics, with underreporting and delayed detections in mountainous regions and areas planted with landmines. Although extensive nationwide interventions, including fencing and depopulation, have been implemented, delays in implementing some measures have generated potential breaches in control strategies, rendering them insufficient for containing transmission. Meanwhile, other measures implemented may have had the unintended effect of facilitating the introduction of the virus in regions unaffected by ASF. ASFV was shown to be self-sustainable in the wild boar population, and expanded from the northwest to southeast following mountain ranges, accelerated by human-mediated viral translocation generating long-distance transmission events. Therefore, ASFV represents a nationwide threat in South Korea with a permanent risk of spillover between wild boar and domestic pig farms. Currently, in the domestic compartment, about half of the 261 farms whose pigs were slaughtered in 2019 were still restricted from restocking pigs due to potential ASF risk from wild boar, and sporadic ASF cases still continue to be reported [15]. It appears essential to understand the transmission dynamics in the wild compartment and spillover to the domestic compartment to formulate cost-effective, risk-based ASF control policies in South Korea. In this discussion, we will build on this synthesis of epidemiological understanding to make recommendations for future research directions.

### 4.1. To Characterize Disease Transmission in Wild Boar

The early stage of the epidemic on pig farms showed between-farm transmission through potentially contaminated vehicles as the main driver of outbreaks. However, this may no longer be the case now that wild boar populations represent an important and constant source of infection in several parts of the country. Therefore, to gain a comprehensive understanding of the transmission dynamics of ASFV, we need to characterize the underlying disease dynamics in wild boar. Due to the limited efficacy of the surveillance system for wild boar, it is likely that cases go undetected and that the spatiotemporal distribution of detected ASF cases reflects potentially heterogeneous surveillance intensity. Together, these two factors are hindering us from unravelling the true underlying ASF dynamics. Successfully unravelling the underlying disease dynamics in wild boar would make it possible to quantify the spatial range of wild boar-mediated transmission, determine the true area of ASF-affected regions, and potentially guide the optimal place and time to implement fencing and depopulation. Despite its implications on wild boar ASF epidemiology, only three studies out of ten for wild boar adjusted or took into account the performance of the surveillance system to understand disease distribution or disease dynamics [32, 36, 38].

The other element that should be considered is the transition of ASF epidemiology. During the past three years of ASFV circulation in South Korea, it is most likely that wild boar population dynamics were highly disturbed, particularly in ASF-affected regions, as their population density has decreased due to high ASF-induced case fatality and intensive depopulation measures [32, 53]. The population dynamics, and therefore disease dynamics, thus are expected to have been substantially changed [54]. For example, in low-density wild boar populations where there are reduced contacts between live wild boar, and thus less direct transmission, carcass-based transmission gradually has had a substantial impact on the disease dynamics due to high viral environmental resistance in infectious carcasses [42, 55]. It is therefore likely that as the disease dynamics progress, the density of wild boar populations will decrease, and the contribution of ASF-positive carcasses to the dynamics may become dominant. Likewise, compared to already affected regions, newly ASF-affected zones, and hence less disturbed regions, are expected to show different disease dynamics. Thus, it is necessary to better understand how ASF epidemiology has changed over time and what factors are responsible for this change. The front wave of ASFV expansion also should be considered separately, and an understanding of the drivers of ASF transmission in wild boar in already affected regions should be updated.

### 4.2. To Understand Ecological Drivers of Disease Distribution and Spread

Seventy percent of South Korea's land is mountainous and covered with forest, making the landscape characteristics quite homogeneous. Most of the forested mountains are located in the eastern section of the Korean peninsula, which has a “east high-west low” geography. In particular, the Taebaek mountain ranges in the east of the country are less fragmented, with sparse road infrastructure and rivers [38], and run from north to south, acting as potential corridors for wild boar migration across the country [25, 32, 37]. This is in contrast to past ASF epidemics, where mountains were thought to be natural barriers for wild boar migration [56]. This mountainous and forested landscape is difficult for humans to navigate, leading to delays in the detection and control of ASFV that allow the virus to spread unimpeded. Due to its current (as of August 2022) spread along the mountain ranges, ASF is expected to be showing different disease dynamics than in the past. However, it is not well-known which characteristics of disease dynamics are different, whether the known risk factors interact with unknown factors, and consequently, how overall disease dynamics are changing. Thus, it is crucial to examine disease dynamics, and update the role of environmental factors on the dynamics and surveillance efficacy. Better knowledge of these will enable us to conduct an ASF risk assessment and prioritize ASF surveillance areas in wild boar.

In South Korea, the identified seasonal peaks of ASF cases in wild boar were only hypothesized to be due to the wild boar's seasonal activities [36]. Apart from the wild boar ecology, there are still various factors that should be taken into account, such as seasonal variation of surveillance intensity and efficacy, meteorological factors, different viral survival, and carcass degradation dependency on the distinct seasonal climate differences in the country [55, 57]. Korean climate is extremely seasonal: summers are usually characterized by high temperatures (between 23°C and 27°C in August on average) and high levels of concentrated annual rainfall, with more than half of the annual precipitation falling during the one-month rainy season in summer, whereas winters are cold (between −6°C and 7°C in January on average) and dry with less than 10% of total annual precipitation [58]. This dry and cold winter prompts wild boar to be more active than during summer [59, 60] due to thermoregulation [60] or feed seeking, thus increasing the contact rate among them. This dry condition also slows down the decomposition of the wild boar' carcasses [61], prolonging the infectious period of ASF-positive carcasses. Also, cold environments increase ASFV survival in carcasses [62], thereby increasing the probability of infection given contact. All of these factors can exacerbate the seasonality of ASF transmission dynamics in wild boar. We acknowledge the challenges in identifying the drivers for seasonality because of their interdependent characteristics. However, clarifying seasonality would help to deploy the limited resources for disease management by providing a clear indicator of when the risk of disease is increasing [54].

Non-wild boar contributions to ASF transmission dynamics have also been highlighted and need to be investigated in the Korean context. Depending on the density of the farms, mechanical vectors are suspected to contribute to promoting transmission [63, 64]. The quantitative distribution of the vectors and their seasonal variation are knowledge gaps that need to be filled. Although the well-known biological vector of ASFV (*Ornithodoros moubata, Ornithodoros erraticus*) has not been reported in South Korea [48], other *Ornithodoros*. spp. are known to be present [65]. These species should be assessed to be potential biological vectors in the wildlife compartment. Regarding scavengers' contributions to ASF transmission dynamics, we did not find any evidence in the literature to support this transmission route. One of the reasons may be that many large carnivores, including gray wolf (*Canis lupus*), dhole or red dog (*Cuon alpinus*), red fox (*Vulpes vulpes*), brown bear (*Ursus arctos*), Asiatic black bear (*Ursus thibetanus*), lynx (*Lynx lynx*), leopard cat (*Prionailurus bengalensis*), leopard (*Panthera pardus*), and Siberian tiger (*Panthera tigris*), became extinct or endangered due to habitat loss, hunting, and human-wildlife conflicts [66]. Thus, the small scavengers, such as raccoon dogs (*Nyctereutes procyonoides*) and ravens (*Corvus corone*), became the main species that come into contact with carcasses of wild boar and domestic pigs, as observed in field experiments in South Korea [67]. These small wildlife species are unlikely to move pig's or wild boar's carcasses to other locations as they usually take small bites from the carcass [61]. This suggests a limited role of scavengers in ASF dynamics in South Korea.

### 4.3. To Assess Social and Psychological Factors Related to ASF Management

We found almost no information on the influence of human behaviours and socioeconomic determinants on disease transmission and management. Although hunting and self-participatory surveillance activities by the stakeholders involved in wild boar were considered to be responsible for the long-distance dispersal of ASFV in South Korea, it is unclear how well the stakeholders understand, comply with, and practice various individual biosecurity measures, what kind of behaviours are perceived to be risky, and how to manage them. For the domestic compartment, there is a rather unclear understanding of farmers' behaviour as an indirect pathway of ASFV incursion into farms. Domestic pig farms in South Korea are usually indoor commercial farms with on-farm biosecurity measures, restricting direct contact with wildlife. Additionally, given that most ASF-positive domestic pigs were found in gestation or parturition pigsties [30], which are supposed to be visited frequently by farmers, human behaviours are likely to be mainly responsible for ASFV transmission to domestic pigs from ASFV sources. In particular, farmers' activities in agricultural land where human-wild boar interaction has been identified in South Korea [68] should be taken into consideration, as suggested in other contexts [69]. Future research studies should characterize stakeholders' behaviours, investigate their roles in human-mediated viral translocation in wild boar and on pig farms, and examine their perception of disease risk.

The Korean government has mandated strict on-farm biosecurity on domestic pig farms, such as outer and inner fences, separated human driveways, separated storage rooms, structured loading chutes, anterooms, and separated dead stock areas, in an effort to prevent ASF outbreaks [70]. While studies have identified the various on-farm biosecurity measures associated with ASF occurrence in various contexts [71, 72], these measures need to be better adapted to the Korean context because their contribution to ASF occurrence may differ depending on the local context. For example, fencing was identified as effective for outdoor pig farms in a specific context, but is less likely to be applicable to the Korean context [2]. The mandatory legislation mentioned at the beginning of this paragraph may put financial and psychological strains on farmers, particularly smallholders. Even though the South Korean government plans to provide financial support for the implementation of the mandatory measures, it is still necessary to comprehend not only the optimal amount and extent of compensation, but also how other psychosocial, economic, and cultural factors interact with these on-farm biosecurity measures and influence the decision-making process on biosecurity behaviours. Better knowledge of these factors and their relationships can improve biosecurity behaviours and help to prevent human-mediated viral translocation while also easing stakeholders' burdens. Along the same line, in addition to reducing the risk of ASF occurrence, evidence-based on-farm biosecurity should also be elicited to strengthen the farm's overall health system, which should thus promote on-farm population health and ease stakeholder burdens.

The likelihood of reporting suspicious cases of ASF is likely to heavily depend on socioeconomic factors as well as disease knowledge. Indeed, in the domestic compartment, reporting rates by farmers has been shown to be affected by social stigma, cultural characteristics, tradition, trust in animal health authorities, and economic aspects [73, 74]. For the stakeholders potentially notifying wildlife cases, the reporting of wild boar carcasses was considered to be driven by ethics or financial incentives, dependent on the context [75]. In addition, stakeholders' views on ASF control measures, such as fencing and zoning policies, can be poles apart [76, 77], which might affect compliance and thus the efficacy of the measures implemented. These factors and the opinions of the stakeholders should be clarified to improve cooperation with them.

### 4.4. To Develop Tools to Identify Effective Management Strategies

The South Korean government has followed the approaches that resulted in successful ASF eradication of wild boar in Belgium and the Czech Republic. However, the fencing approach in South Korea was evaluated as being less effective in blocking ASFV expansion and ASFV kept being reported on the other side of the fences. Several factors could explain this inconsistent outcome. First of all, the current South Korean ASF epidemic in wild boar is probably due to more than one focal introduction [31] or due to continuous infection pressure along the national border [32]. This is obviously a different situation compared with Belgium and the Czech Republic, where single focal introductions were highly suspected to be the source of ASFV [23]. Second, several medium-to-long-distance, human-mediated viral translocation was likely to have occurred relatively early in the epidemic, resulting in a very wide front wave and jeopardizing the effectiveness of the policies implemented [31]. Third, the different outcomes might be explained by the geographical specificities of the country, which is principally covered by forests and mountains that provide favourable conditions for wild boar while making surveillance and control difficult [32, 36]. Indeed, low human accessibility to outbreak regions where some areas are prohibited or limited due to civilian control zone, landmines, Korean Demilitarized Zone, and rough landscape characteristics [32] hampered timely situational awareness. The “east high-west low” geography of the Korean peninsula may contribute to delayed implementations of management measures in the east region. This hypothesis is supported by current ASF transmission dynamics, in which the ASFV epidemic originating in northwest regions was confined, while the other originating from northern regions has invaded southeast regions [31]. Fourth, another factor could be the delays observed in erecting fences in some places, potentially creating breaches in the control scheme and allowing the virus to spread. In some counties, the deployment of the decision took longer than it should have due to administrative delays [31].

To provide an effective management strategy to policymakers, we first need to improve our understanding of the effects of the measures implemented on disease dynamics. The fences to prevent ASFV dispersion may interact with landscape features, as can be extrapolated from wildlife ecology studies demonstrating that watercourses make fences vulnerable to wildlife crossing [78, 79]. The estimated limited effects of fencing on ASF transmission in South Korea may reflect this landscape-dependent heterogeneity. Likewise, hunting and trapping might lead to positive or negative consequences on ASF transmission depending on the landscape features or wild boar density [80, 81]. In particular, as hunting is a non-silent form of culling compared to trapping, it may create more disturbances in wild boar populations and provoke behavioural changes. Consequently, additional studies are required to assess the effectiveness of these different control measures and to identify the environmental factors that affect them. It would be possible to create a map that shows the effects of control measures, and the map could be paired with a risk map of spatial invasion to determine the optimal allocation of control measures.

Finally, we need tools to be able to characterize ongoing ASF outbreak dynamics in real-time and forecast what might happen if various control measures are implemented, while comprehensively accounting for the knowledge gaps discussed above, including undetected ASF in wild boar, changing epidemiology, ecological drivers, impacts of human behaviour, and heterogeneous effects of control measures. Moreover, to provide more evidence to policymakers, the tools should also reflect the feasibility of the control measures represented as a function of the resources of the animal health authorities and the magnitude of the disease [20, 82]. These tools would improve situational awareness and inform the optimized response to rapidly changing disease dynamics, which was needed but absent in past outbreaks. For wild compartments, these can be used to assess the current ASF-affected areas, and thus where and when to install fences. For the domestic compartment, these would guide the ASF exit strategy to restock pigs for newly ASF-positive farms as well as the ones that have been banned from operating since 2019. These could be highly useful not only in South Korea but in other countries affected or threatened by ASF as well. There was only one transmission model of ASF in wild boar in South Korea [38], thus there is a need to develop complementary models using different paradigms to provide more evidence-based recommendations to policymakers [20].

## 5. Conclusion

In this review, we highlighted the current understanding of ASF epidemiology in South Korea. Although the ASF outbreaks in domestic pig farms were contained successfully, there is a continuing spillover risk due to the self-sustaining ASFV transmission in wild boars despite extensive national control policies, indicating that control policies targeting wild boar should be strengthened. We laid out future research directions that could help to understand ASFV transmission dynamics within wild boar populations and spillover events to domestic pig farms so as to identify optimal control policies in both populations. We believe that this research will contribute to filling knowledge gaps and provide useful recommendations for effective policies that could ultimately contribute to the eradication of ASF in South Korea.

## Figures and Tables

**Table 1:** Included studies and their target host.

Author	Year	Title	Species
Jo and Gortazar [26]	2020	African swine fever in wild boar, South Korea, 2019	WB
Yoon et al. [27]	2020	Clinical symptoms of African swine fever in domestic pig farms in the Republic of Korea, 2019	DP
Cho et al. [28]	2021	Quantitative risk assessment of the African swine fever introduction into the Republic of Korea via legal import of live pigs and pig products	PP
Cho et al. [29]	2020	Population viability analysis to estimate the needed number of capture-and-remove wild boars for control of African swine fever in the Republic of Korea	WB
Cho et al. [30]	2021	Surveillance of ASF-infected pig farms from September to October 2019 in South Korea	DP
Jo and Gortazar [31]	2021	African swine fever in wild boar: assessing interventions in South Korea	WB
Lim et al. [32]	2021	Modelling the spatial distribution of ASF-positive wild boar carcasses in South Korea using 2019 F02D 2020 national surveillance data	WB
Kim and Pak [33]	2021	Estimating the basic reproduction number for the 2019/20 African swine fever epidemics in wild boar in the Republic of Korea	WB
Yoon et al. [34]	2021	Arthropods as potential vectors of African swine fever virus outbreaks in pig farms in the Republic of Korea	Arthropod
Yoo et al. [35]	2021	Transmission dynamics of African swine fever virus, South Korea, 2019	DP
Lim et al. [36]	2021	Basic reproduction number of African swine fever in wild boar (*Sus scrofa*) and its spatiotemporal heterogeneity in South Korea	WB
Ko et al. [37]	2021	The analysis of African swine fever disease spread using agent-based model	WB
Han et al. [38]	2021	Understanding the transmission of African swine fever in wild boars of South Korea: a simulation study for parameter estimation	WB
Lim et al. [25]	2022	Prediction of potential spread areas of African swine fever virus through wild boars using maxent model	WB

PP: pig products; WB: wild boar; and DP; domestic pigs.

## Data Availability

The data used in the analyses can be obtained from the corresponding author upon reasonable request.

## References

[1] Sanchez-Vizcaino J. M., Mur L., Gomez-Villamandos J., Carrasco L. (2015). An update on the epidemiology and pathology of African swine fever. *Journal of Comparative Pathology*.

[2] Dixon L. K., Stahl K., Jori F., Vial L., Pfeiffer D. U. (2020). African swine fever epidemiology and control. *Annual Review of Animal Biosciences*.

[3] Guberti V., Kerba S., Masiulis M., Khomenko S. (2022). *African Swine Fever in Wild Boar – Ecology and Biosecurity*.

[4] Chenais E., Depner K., Guberti V., Dietze K., Viltrop A., Stahl K. (2019). Epidemiological considerations on African swine fever in Europe 2014-2018. *Porcine Health Management*.

[5] Kim H. J., Cho K., Lee S. (2020). Outbreak of African swine fever in South Korea, 2019. *Transboundary and Emerging Diseases*.

[6] Vergne T., Guinat C., Pfeiffer D. U. (2020). Undetected circulation of African swine fever in wild boar, Asia. *Emerging Infectious Diseases*.

[7] Kim H. J., Lee M. J., Lee S. K. (2019). African swine fever virus in pork brought into South Korea by travelers from China, August 2018. *Emerging Infectious Diseases*.

[8] Ministry of Agriculture (2018). African swine fever prevention and management measures. https://www.mafra.go.kr/home/5109/subview.do?enc=Zm5jdDF8QEB8JTJGYmJzJTJGaG9tZSUyRjc5MiUyRjU1OTk1NCUyRmFydGNsVmlldy5kbyUzRmJic0NsU2VxJTNEJTI2cmdzRW5kZGVTdHIlM0QyMDIwLjEwLjI4JTI2YmJzT3BlbldyZFNlcSUzRCUyNnBhc3N3b3JkJTNEJTI2c3JjaENvbHVtbiUzRHNqJTI2cGFnZSUzRDI3JTI2cmdzQmduZGVTdHIlM0QyMDE4LjAxLjAxJTI2cm93JTNEMTAlMjZpc1ZpZXdNaW5lJTNEZmFsc2UlMjZzcmNoV3JkJTNEJUVDJTk1JTg0JUVEJTk0JTg0JUVCJUE2JUFDJUVDJUI5JUI0JTI2.

[9] Kim Y. J., Park B., Kang H. E. (2021). Control measures to African swine fever outbreak: active response in South Korea, preparation for the future, and cooperation. *Journal of Veterinary Science*.

[10] Yoo D., Kim H., Lee J. Y., Yoo H. S. (2020). African swine fever: etiology, epidemiological status in Korea, and perspective on control. *Journal of Veterinary Science*.

[11] Ministry of Agriculture (2022). Status of livestock disease occurrence (African swine fever). https://www.mafra.go.kr/FMD-AI2/map/ASF/ASF_map.jsp.

[12] Ministry of Environments (2021). Status of wild boar ASF (African swine fever) outbreak in Korea. http://www.me.go.kr/home/web/policy_data/read.do?menuId=10261&seq=7815.

[13] Ministry of Agriculture (2019). Major control measures for African swine fever. https://www.mafra.go.kr/home/5109/subview.do?enc=Zm5jdDF8QEB8JTJGYmJzJTJGaG9tZSUyRjc5MiUyRjU2MTQ3MCUyRmFydGNsVmlldy5kbyUzRmJic0NsU2VxJTNEJTI2cmdzRW5kZGVTdHIlM0QyMDE5LjEyLjMxJTI2YmJzT3BlbldyZFNlcSUzRCUyNnBhc3N3b3JkJTNEJTI2c3JjaENvbHVtbiUzRHNqJTI2cGFnZSUzRDYlMjZyZ3NCZ25kZVN0ciUzRDIwMTguMDEuMDElMjZyb3clM0QxMCUyNmlzVmlld01pbmUlM0RmYWxzZSUyNnNyY2hXcmQlM0QlRUMlOTUlODQlRUQlOTQlODQlRUIlQTYlQUMlRUMlQjklQjQlMjY%3D.

[14] Ministry of Agriculture (2021). African swine fever standard operating procedure (SOP). https://www.mafra.go.kr/home/5109/subview.do?enc=Zm5jdDF8QEB8JTJGYmJzJTJGaG9tZSUyRjc5MiUyRjU2MjQ4NSUyRmFydGNsVmlldy5kbyUzRmJic0NsU2VxJTNEJTI2cmdzRW5kZGVTdHIlM0QyMDIwLjEwLjI4JTI2YmJzT3BlbldyZFNlcSUzRCUyNnBhc3N3b3JkJTNEJTI2c3JjaENvbHVtbiUzRHNqJTI2cGFnZSUzRDQlMjZyZ3NCZ25kZVN0ciUzRDIwMjAuMDIuMDElMjZyb3clM0QxMCUyNmlzVmlld01pbmUlM0RmYWxzZSUyNnNyY2hXcmQlM0QlRUMlOTUlODQlRUQlOTQlODQlRUIlQTYlQUMlRUMlQjklQjQlMjY%3D.

[15] Ministry of Agriculture Food and Rural Affairs (2020). Ministry of agriculture, food and rural affairs. https://www.mafra.go.kr/home/5109/subview.do?enc=Zm5jdDF8QEB8JTJGYmJzJTJGaG9tZSUyRjc5MiUyRjU2MjQ4NSUyRmFydGNsVmlldy5kbyUzRnJnc0JnbmRlU3RyJTNEMjAxOS4wNi4wMSUyNmlzVmlld01pbmUlM0RmYWxzZSUyNnJnc0VuZGRlU3RyJTNEMjAyMC4xMi4xMSUyNmJic09wZW5XcmRTZXElM0QlMjZwYWdlJTNENCUyNnJvdyUzRDEwJTI2cGFzc3dvcmQlM0QlMjZiYnNDbFNlcSUzRCUyNnNyY2hDb2x1bW4lM0RzaiUyNnNyY2hXcmQlM0QlRUMlOTUlODQlRUQlOTQlODQlRUIlQTYlQUMlRUMlQjklQjQlMjY%3D.

[16] Anonymous (2021). Act on the prevention of contagious animal diseases. https://elaw.klri.re.kr/kor_service/lawView.do?hseq=55242&lang=ENG.

[17] Ministry of Agriculture (2021). Special control measures due to the spread of wild boar ASF-infected areas. https://me.go.kr/home/web/board/read.do?pagerOffset=120&maxPageItems=10&maxIndexPages=10&searchKey=&searchValue=&menuId=&orgCd=&boardId=1424800&boardMasterId=1&boardCategoryId=39&decorator=.

[18] Ministry of Environments (2021). Measures to block the spread of wild boar African swine fever in winter. http://www.me.go.kr/home/web/board/read.do;jsessionid=ZY2twW6-6QyHbeblT6bSV2J9.mehome1?pagerOffset=20&maxPageItems=10&maxIndexPages=10&searchKey=content&searchValue=%EC%9A%B8%ED%83%80%EB%A6%AC&menuId=286&orgCd=&boardId=1421820&boardMasterId=1&boardCategoryId=&decorator=.

[19] Brookes V. J., Barrett T. E., Ward M. P. (2021). A scoping review of African swine fever virus spread between domestic and free-living pigs. *Transbound Emerg Dis*.

[20] Hayes B. H., Andraud M., Salazar L. G., Rose N., Vergne T. (2021). Mechanistic modelling of African swine fever: a systematic review. *Preventive Veterinary Medicine*.

[21] Danzetta M. L., Marenzoni M. L., Iannetti S., Tizzani P., Calistri P., Feliziani F. (2020). African swine fever: lessons to learn from past eradication experiences. A systematic review. *Frontiers in Veterinary Science*.

[22] Guinat C., Gogin A., Blome S. (2016). Transmission routes of African swine fever virus to domestic pigs: current knowledge and future research directions. *The Veterinary Record*.

[23] Sauter-Louis C., Schulz K., Richter M., Staubach C., Mettenleiter T. C., Conraths F. J. (2021). African swine fever: why the situation in Germany is not comparable to that in the Czech Republic or Belgium. *Transboundary and Emerging Diseases*.

[24] Tricco A. C., Lillie E., Zarin W. (2018). PRISMA extension for scoping reviews (PRISMA-ScR): checklist and explanation. *Annals of Internal Medicine*.

[25] Lim S. J., Namgung H., Kim N. H., Oh Y., Park Y. C. (2022). Prediction of potential spread areas of African swine fever virus through wild boars using Maxent model. *Journal of Ecology and Environment*.

[26] Jo Y. S., Gortazar C. (2020). African swine fever in wild boar, South Korea. *Transboundary and Emerging Diseases*.

[27] Yoon H., Hong S. K., Lee I. (2020). Clinical symptoms of African swine fever in domestic pig farms in the Republic of Korea. *Transboundary and Emerging Diseases*.

[28] Cho K. H., Kim H., Kim Y., Kang H., Martinez‐Lopez B., Lee J. (2021). Quantitative risk assessment of the African swine fever introduction into the Republic of Korea via legal import of live pigs and pig products. *Transboundary and Emerging Diseases*.

[29] Cho H.-K., Jung B.-S., Jung C.-S., Kim E.-T., Pak Son I. (2020). Population viability analysis to estimate the needed number of capture-and-remove wild boars for control of African swine fever in the Republic of Korea. *Journal of the Preventive Veterinary Medicine*.

[30] Cho K. H., Kim H. J., Kim D. Y. (2021). Surveillance of ASF-infected pig farms from september to october 2019 in South Korea. *Journal of Veterinary Science*.

[31] Jo Y. S., Gortázar C. (2021). African swine fever in wild boar: assessing interventions in South Korea. *Transboundary and Emerging Diseases*.

[32] Lim J. S., Vergne T., Pak S. I., Kim E. (2021). Modelling the spatial distribution of asf‐positive wild boar carcasses in South Korea using 2019–2020 national surveillance data. *Animals*.

[33] Kim E.-T., Pak S.-I. (2021). Estimating the basic reproduction number for the 2019/20 African swine fever epidemics in wild boars in the Republic of Korea. *Journal of the Preventive Veterinary Medicine*.

[34] Yoon H., Hong S., Lee I. (2021). Arthropods as potential vectors of African swine fever virus outbreaks in pig farms in the Republic of Korea. *Veterinary Medical Science*.

[35] Yoo D. S., Kim Y., Lee E. S. (2021). Transmission dynamics of african swine fever virus, South Korea. *Emerging Infectious Diseases*.

[36] Lim J. S., Kim E., Ryu P. D., Pak S. I. (2021). Basic reproduction number of African swine fever in wild boars (*Sus scrofa*) and its spatiotemporal heterogeneity in South Korea. *Journal of Veterinary Science*.

[37] Ko C., Cho W., Hwang B., Ko D. W., Kang W. (2021). The analysis of african swine fever disease spread using agent-based model. *Journal of the Korean Cadastre Information Association*.

[38] Han J. H., Yoo D. S., Pak S. I., Kim E. T. (2021). Understanding the transmission of African swine fever in wild boars of South Korea: a simulation study for parameter estimation. *Transboundary and Emerging Diseases*.

[39] European Food Safety Authority Efsa, Anette B., Anette B. (2020). Epidemiological analyses of African swine fever in the European union (November 2018 to October 2019). *EFSA Journal*.

[40] Yoo D. S., Chun B.Y., Lee K. N., Moon O. K. (2021). Dynamics of inter-farm transmission of highly pathogenic avian influenza H5N6 integrating vehicle movements and phylogenetic information. *Scientific Reports*.

[41] Yoo D. S., Song Y., Choi D., Lim J., Lee K., Kang T. (2022). Machine learning-driven dynamic risk prediction for highly pathogenic avian influenza at poultry farms in Republic of Korea: daily risk estimation for individual premises. *Transboundary and Emerging Diseases*.

[42] Pepin K. M., Golnar A. J., Abdo Z., Podgorski T. (2020). Ecological drivers of African swine fever virus persistence in wild boar populations: insight for control. *Ecology and Evolution*.

[43] European Food Safety Authority Efsa, Desmecht D., Gerbier G. (2021). Epidemiological analysis of African swine fever in the European union (September 2019 to August 2020). *EFSA Journal*.

[44] Dellicour S., Desmecht D., Paternostre J. (2020). Unravelling the dispersal dynamics and ecological drivers of the African swine fever outbreak in Belgium. *Journal of Applied Ecology*.

[45] European Food Safety Authority (2020). Epidemiological analyses of African swine fever in the European Union.

[46] European Food Safety Authority Efsa, Nielsen S. S., Alvarez J. (2021). Research priorities to fill knowledge gaps in the control of African swine fever: possible transmission of African swine fever virus by vectors. *EFSA Journal*.

[47] Saegerman C., Bonnet S., Bouhsira E. (2021). An expert opinion assessment of blood-feeding arthropods based on their capacity to transmit African swine fever virus in Metropolitan France. *Transboundary and Emerging Diseases*.

[48] Chae J. B., Kang J. G., Kim H. C. (2017). Identification of tick species collected from wild boars and habitats of wild boars and domestic pigs in the Republic of Korea. *Korean Journal of Parasitology*.

[49] Baldacchino F., Muenworn V., Desquesnes M., Desoli F., Charoenviriyaphap T., Duvallet G. (2013). Transmission of pathogens by Stomoxys flies (Diptera, Muscidae): a review. *Parasite*.

[50] Lloyd-Smith J. O., Cross P. C., Briggs C. J. (2005). Should we expect population thresholds for wildlife disease?. *Trends in Ecology & Evolution*.

[51] Mysterud A., Rolandsen C. M., McCallum H. (2018). Fencing for wildlife disease control. *Journal of Applied Ecology*.

[52] Mysterud A., Rauset G. R., Van Moorter B., Andersen R., Strand O., Rivrud I. M. (2020). The last moves: the effect of hunting and culling on the risk of disease spread from a population of reindeer. *Journal of Applied Ecology*.

[53] Morelle K., Bubnicki J., Churski M., Gryz J., Podgorski T., Kuijper D. P. J. (2020). Disease-induced mortality outweighs hunting in causing wild boar population crash after african swine fever outbreak. *Frontiers in Veterinary Science*.

[54] Bergmann H., Schulz K., Conraths F. J., Sauter-Louis C. (2021). A review of environmental risk factors for african swine fever in European wild boar. *Animals*.

[55] Podgorski T., Borowik T., Lyjak M., Wozniakowski G. (2020). Spatial epidemiology of African swine fever: host, landscape and anthropogenic drivers of disease occurrence in wild boar. *Preventive Veterinary Medicine*.

[56] Cwynar P., Stojkov J., Wlazlak K. (2019). African swine fever status in Europe. *Viruses*.

[57] O’Neill X., White A., Ruiz-Fons F., Gortazar C. (2020). Modelling the transmission and persistence of African swine fever in wild boar in contrasting European scenarios. *Scientific Reports*.

[58] Adminstration K. M. (2023). Climate of Korea. https://www.kma.go.kr/eng/biz/climate_01.jsp.

[59] Johann F., Handschuh M., Linderoth P., Dormann C. F., Arnold J. (2020). Adaptation of wild boar (*Sus scrofa*) activity in a human-dominated landscape. *BMC Ecology*.

[60] Lemel J., Truvé J., Söderberg B. (2003). Variation in ranging and activity behaviour of European wild boar *Sus scrofa* in Sweden. *Wildlife Biology*.

[61] Probst C., Gethmann J., Amler S., Globig A., Knoll B., Conraths F. J. (2019). The potential role of scavengers in spreading African swine fever among wild boar. *Scientific Reports*.

[62] Fischer M., Huhr J., Blome S., Conraths F. J., Probst C. (2020). Stability of african swine fever virus in carcasses of domestic pigs and wild boar experimentally infected with the ASFV “Estonia 2014” isolate. *Viruses*.

[63] Bonnet S. I., Bouhsira E., De Regge N. (2020). Putative role of arthropod vectors in african swine fever virus transmission in relation to their bio-ecological properties. *Viruses*.

[64] Vergne T., Andraud M., Bonnet S. (2021). Mechanical transmission of African swine fever virus by Stomoxys calcitrans: insights from a mechanistic model. *Transboundary and Emerging Diseases*.

[65] Han S. W., Chae J. B., Jo Y. S. (2020). First report of newly identified Ornithodoros species in the Republic of Korea. *The Journal of Parasitology*.

[66] Won C., Smith K. G. (1999). History and current status of mammals of the Korean Peninsula. *Mammal Review*.

[67] Cho H.-K., Kim E. T., Jung B. S., Pak S. I. (2021). A preliminary investigation into the decomposition rate of wild boar carcasses in forest habitats. *Journal of the Preventive Veterinary Medicine*.

[68] Lee S. M., Lee E. J. (2019). Diet of the wild boar (*Sus scrofa*): implications for management in forest-agricultural and urban environments in South Korea. *PeerJ*.

[69] European Food Safety Authority Efsa, Boklund A., Cay B. (2018). Epidemiological analyses of african swine fever in the European union (november 2017 until november 2018). *EFSA Journal*.

[70] Ministry of Agriculture Food and Rural Affairs (2022). Ministry of agriculture, food and rural affairs. https://www.mafra.go.kr/home/5109/subview.do?enc=Zm5jdDF8QEB8JTJGYmJzJTJGaG9tZSUyRjc5MiUyRjU2NDI3OCUyRmFydGNsVmlldy5kbyUzRnJnc0VuZGRlU3RyJTNEMjAyMi4wNy4zMSUyNmJic09wZW5XcmRTZXElM0QlMjZwYWdlJTNEMSUyNnJvdyUzRDEwJTI2cGFzc3dvcmQlM0QlMjZyZ3NCZ25kZVN0ciUzRDIwMjIuMDUuMjQlMjZiYnNDbFNlcSUzRCUyNnNyY2hDb2x1bW4lM0RzaiUyNmlzVmlld01pbmUlM0RmYWxzZSUyNnNyY2hXcmQlM0QlRUMlOEIlOUMlRUQlOTYlODklRUElQjclOUMlRUMlQjklOTklMjY%3D.

[71] Bellini S., Casadei G., De Lorenzi G., Tamba M. (2021). A review of risk factors of african swine fever incursion in pig farming within the European union scenario. *Pathogens*.

[72] Bergmann H., Dups-Bergmann J., Schulz K. (2022). Identification of risk factors for african swine fever: a systematic review. *Viruses*.

[73] Gates M. C., Earl L., Enticott G. (2021). Factors influencing the performance of voluntary farmer disease reporting in passive surveillance systems: a scoping review. *Preventive Veterinary Medicine*.

[74] Vergne T., Guinat C., Petkova P. (2016). Attitudes and beliefs of pig farmers and wild boar hunters towards reporting of African swine fever in Bulgaria, Germany and the western part of the Russian federation. *Transbound Emerg Dis*.

[75] Urner N., Sauter-Louis C., Staubach C., Conraths F. J., Schulz K. (2021). A comparison of perceptions of Estonian and Latvian hunters with regard to the control of african swine fever. *Frontiers in Veterinary Science*.

[76] Urner N., Motus K., Nurmoja I. (2020). Hunters’ acceptance of measures against African swine fever in wild boar in Estonia. *Preventive Veterinary Medicine*.

[77] Moskalenko L., Schulz K., Motus K., Viltrop A. (2022). Pigkeepers’ knowledge and perceptions regarding African swine fever and the control measures in Estonia. *Preventive Veterinary Medicine*.

[78] Laguna E., José A. B., Antonio J. C., Joaquín V., Pelayo A. (2022). Permeability of artificial barriers (fences) for wild boar (*Sus scrofa*) in Mediterranean mixed landscapes. *Pest Management Science*.

[79] Cozzi G., Broekhuis F., McNutt J. W., Schmid B. (2013). Comparison of the effects of artificial and natural barriers on large African carnivores: implications for interspecific relationships and connectivity. *Journal of Animal Ecology*.

[80] Jori F., Massei G., Licoppe A. (2021). Management of wild boar populations in the European Union before and during the ASF crisis. *Understanding and Combatting African Swine Fever: A European Perspective*.

[81] Salazar L. G., Rose N., Hayes B. (2022). Effects of habitat fragmentation and hunting activities on African swine fever dynamics among wild boar populations. *Preventive Veterinary Medicine*.

[82] Bozzani F. M., Vassall A., Gomez G. B. (2021). Building resource constraints and feasibility considerations in mathematical models for infectious disease: a systematic literature review. *Epidemics*.

